# Supplementary material for: Attachment towards the Owner Is Associated with Spontaneous Sleep EEG Parameters in Family Dogs
Source: Animals (Basel). 2022 Mar 31;12(7):895. doi: 10.3390/ani12070895 (PMC8997010; doi:10.3390/ani12070895)
Supplement: Supplementary file 1 [file animals-12-00895-s001.zip › animals-1639577-supplementary.pdf]

animals

MDPI

Supplementary Materials

Attachment towards the Owner Is Associated with Spontaneous Sleep EEG Parameters in Family Dogs

Cecília Carreiro [1,2,*], Vivien Reicher [1,2,3], Anna Kis [2,4] and Márta Gácsi [2,3]

[1] Doctoral School of Biology, Institute of Biology, ELTE Eötvös Loránd University, 1117 Budapest, Hungary; vivien.reicher@gmail.com
[2] Department of Ethology, Institute of Biology, ELTE Eötvös Loránd University, 1117 Budapest, Hungary; vargane.kis.anna@ttk.mta.hu (A.K.); marta.gacsi@gmail.com (M.G.)
[3] MTA-ELTE Comparative Ethology Research Group, 1117 Budapest, Hungary
[4] Institute of Cognitive Neuroscience and Psychology, Research Centre for Natural Sciences, 1117 Budapest, Hungary
* Correspondence: ceciliacarreiro@gmail.com

Table S1. Dogs' demographic data. All information was provided by the owners, except for the standard adult weight (calculated based on the American Kennel Club database; https://www.akc.org/). In the case of mix breed dogs, the weight is the information provided by the owners. Training status may involve no training supervised by a trainer (none), basic obedience (basic) or trainings such as agility, dummy training, service work or scent work (specific). Some dogs had more than one type of specific training and the information was organized into three general types of training status.

Subject	Breed	Age at the EEG (Months)	Sex	Gender Status	Training Status	Standard Adult Weight (kg)
Ginny	Nova Scotia duck tolling retriever	2.4	female	intact	specific	19.1
Kesu	border terrier	3.3	female	intact	specific	5.7
Zeno	border collie	3.63	male	intact	basic	19
Kosza	tervueren	3.8	male	intact	basic	29
Keeva	rough collie	3.9	female	intact	none	22.5
Lotti	golden retriever	4.6	female	intact	basic	27
Szille	golden retriever	5.3	female	intact	none	27
Rozi	labrador retriever	5.8	female	intact	none	28.1
Fuge	mix	5.9	female	neutered	basic	15
Pesto	boston terrier	6.7	female	neutered	basic	9.3
Vackor	golden retriever	6.8	male	intact	none	31.5
Tango	golden retriever	6.9	male	intact	specific	31.5

Pax	cocker spaniel	7.1	female	intact	basic	12
Lulu	mix	7.3	female	neutered	none	11
Zafi	small munsterlander	7.3	female	intact	specific	24
Puki	American staffordshire terrier	7.6	female	intact	none	22.1
Borka	border collie	7.6	female	neutered	specific	17
Dimi	golden retriever	7.7	male	intact	basic	31.5
Byron	golden retriever	8.4	male	intact	basic	31.5
Akira	labradoodle	9.1	female	neutered	specific	26
Rex	miniature German pinscher	10.2	female	neutered	basic	4
Szilka	tervueren	11.6	female	neutered	basic	25
Rico	border collie	11.7	male	intact	basic	19
Misa	mix	12.2	female	intact	specific	22
Mokka	border collie	14.7	female	neutered	specific	17
Luna	Hungarian vizsla	15.3	female	neutered	basic	23
Mirza	springer spaniel	16.6	female	intact	specific	19.1
Bingo	mix	21.8	male	intact	specific	32
Barack	golden retriever	22.2	female	neutered	specific	27
Apacs	Chinese crested	22.4	male	neutered	specific	6
Nara	mix	27.8	female	neutered	specific	28
Luca	mix	31.1	female	neutered	basic	15
Floyd	golden retriever	35.2	male	intact	specific	31.5
Bran	border collie	61.5	female	neutered	none	17
Walter	golden retriever	68	male	intact	specific	31.5
Nia	border collie	70.6	female	intact	specific	17
Joey	Australian shepherd	73.9	male	neutered	specific	22
Rohan	border collie	84.4	female	neutered	basic	17

Monty	border collie	97.1	male	neutered	none	17
Alma	mix	102.6	female	neutered	basic	22
Barney	golden retriever	104.9	male	intact	basic	31.5
Mini	mix	117.9	female	neutered	specific	18

Note: although differences in brain electrophysiology during sleep has been found related to sexual hormones in dogs (intact vs. neutered females) [1], we did not include gender status in our analysis due to its overlap with age. In our sample size, neutered dogs up to 8 months old corresponded to only 9.5%, while this number increased to 36% of older dogs. Also, breed size has shown differences in brain electrophysiology along cortex maturation, however, we did not consider specifically the breeds for two reasons. First, our sample size had only 4 small breed dogs (e.g., under 10 kg), no giant breeds and our data had no outliers, as observed in [2] for giant breeds. Second, to circumvent measurement error due to the variability among the breeds head musculature and skull shape and thickness, dog EEG data were analyzed using not absolute, but relative EEG power [3] and breed did not need to be used as a separated factor.

Table S2. Means and standard deviations of the variables assessed in dogs tested first on SST (n=19) or first on EEG (n=23). No effect of the order of the tests was found.

Variable	SST First	EEG First
**Attachment scores**	M ± SD: 5.84 ± 2.21	M ± SD: 6.30 ± 2.62
**Drowsiness duration (%)**	M ± SD: 19.9 ± 9.51	M ± SD: 21.4 ± 10.5
**NREM duration (%)**	M ± SD: 34.9 ± 15.2	M ± SD: 37.3 ± 14.6
**REM duration (%)**	M ± SD: 11.4 ± 8.88	M ± SD: 8.10 ± 5.69
**Alpha power (log)**	M ± SD: 0.27 ± 0.15	M ± SD: 0.27 ± 0.14
**Alpha-Delta anticorrelation (log)**	M ± SD: 0.16 ± 0.10	M ± SD: 0.19 ± 0.13

References

1. Iotchev, I.B.; Kis, A.; Turcsán, B.; Tejeda Fernández de Lara, D.R.; Reicher, V.; Kubinyi, E. Age-Related Differences and Sexual Dimorphism in Canine Sleep Spindles. *Sci. Rep.* **2019**, *9*, 1–11. https://doi.org/10.1038/s41598-019-46434-y.
2. Reicher, V.; Bunford, N.; Kis, A.; Carreiro, C.; Csibra, B.; Kratz, L.; Gácsi, M. Developmental Features of Sleep Electrophysiology in Family Dogs. *Sci. Rep.* **2021**, *11*, 22760. https://doi.org/10.1038/s41598-021-02117-1.
3. Kis, A.; Szakadát, S.; Kovács, E.; Gácsi, M.; Simor, P.; Gombos, F.; Topál, J.; Miklósi, Á.; Bódizs, R. Development of a Non-Invasive Polysomnography Technique for Dogs (Canis Familiaris). *Physiol. Behav.* **2014**, *130*, 149–156. https://doi.org/10.1016/j.physbeh.2014.04.004.
